# Experiences of acquired brain injury one-month post-discharge from acute hospitalisation

**DOI:** 10.4102/ajod.v12i0.1037

**Published:** 2023-02-28

**Authors:** Kirsten J. Talbot, Esedra Krüger, Bhavani S. Pillay

**Affiliations:** 1 Department of Speech-Language Pathology and Audiology, Faculty of Humanities, University of Pretoria, Pretoria, South Africa

**Keywords:** acquired brain injury, experiences, acute care, post-hospitalisation, significant others, qualitative research, semi-structured interview

## Abstract

**Background:**

Healthcare professionals may have a preconceived idea about life after an acquired brain injury (ABI). Understanding lived experiences of individuals with ABI and their significant others, post-hospitalisation, may improve communication between healthcare professionals and individuals directly influenced by the ABI.

**Objective:**

To describe perceived experiences of individuals with ABI, and their significant others, regarding rehabilitation services and returning to daily activities, one-month post-discharge from acute hospitalisation.

**Method:**

Semi-structured interviews, via an online platform, expanded on the experiences of six dyads (individuals with an ABI and their significant others). Data were thematically analysed.

**Results:**

Six main themes emerged that best described participants’ experiences; two of which were shared between individuals with ABI and their significant others (SO). Individuals with an ABI acknowledged recovery as their priority and highlighted the importance of patience. The need for counselling and additional support from healthcare professionals and peers arose. The SO expressed a need for written information, improved communication from healthcare professionals, and education regarding the implications of an ABI. The coronavirus disease 2019 (COVID-19) pandemic negatively influenced all participants’ overall experiences, mainly because of termination of visiting hours. Psychosocial intervention would have been beneficial to all participants. Faith influenced most participants’ attitudes towards recovery and adapting post-ABI.

**Conclusion:**

Most participants accepted their new reality but required additional support to cope emotionally. Individuals with an ABI would benefit from opportunities to share experiences with and learn from others in a similar situation. Streamlined services and improved communication may alleviate anxiety among families during this crucial transitional period.

**Contribution:**

This article provides valuable information on the perspectives and experiences of individuals with ABI and their significant others during the transition from acute hospitalisation. The findings can assist with the continuity of care, integrative health and supportive strategies during the transition period post-ABI.

## Introduction

Acquired brain injuries (ABI) include several conditions, most commonly, strokes and traumatic brain injuries (TBIs), which are local and global health concerns (Ntsiea 2019; Menon & Bryant 2019). Strokes affect roughly 240 South Africans daily and is the cause of 25 000 deaths annually (Taylor & Ntusi 2019; The Heart and Stroke Foundation South Africa 2020). Globally, TBIs are estimated to affect 69 million individuals annually, with the most proportion (56%) of TBI’s consequent to road traffic collisions occurring in South Africa and South-east Asia (Dewan et al. 2018). An ABI is diverse in consequence and severity, and can influence one’s motor, sensory and/or communicative functioning, hindering participation in daily activities (Chembeni & Nkomo 2017; Webster, Taylor & Balchin 2015). Individuals with an ABI are treated by a healthcare team, in private and public care settings, to assist with education and understanding of the ABI within the International Classification of Functioning, Disability and Health framework (ICF) (Degeneffe 2015; Ntsiea 2019). The significant other (SO), such as a family member, close relative or caregiver, who cares for the individual with an ABI is unprepared and often feels ill-equipped to meet the resultant changes that occur in their loved one after an ABI (Degeneffe 2015; Whitehead & Baalbergen 2019).

Individuals with ABI are likely to experience difficulties in all areas of the ICF, especially interaction between themselves and their environment (Kusambiza-Kiingi, Maleka & Ntsiea 2017; Ntsiea 2019). Difficulties seen during the early phase of recovery may include loss of memory, loss of physical functioning and difficulty verbally communicating, hindering active participation in daily activities (Kusambiza Kiingi et al. 2017; Stiekema et al. 2020). Experiences within the first week until the first month post-ABI are critical because of the transition periods from hospitalisation, rehabilitation, and reintegration into the community (Ntsiea 2019). This includes access to information regarding education about an ABI, caregiver education and discharge readiness before discharge from acute care and during the ongoing rehabilitation process (Piccenna et al. 2016; Stiekema et al. 2020).

Providing information to individuals with ABI and their family is important; however, communication between healthcare professionals (HCP) and family members has been recorded as ‘inadequate’, resulting in poor understanding and implementation of health information (Soeker & Ganie 2019). In cases where SOs have received information about an ABI, they report pamphlets as difficult to understand and are hesitant to ask for further explanations (Li et al. 2020; Webster et al. 2015). There is a significant need for HCP to better understand the social context of individuals with ABI while in the acute care setting and during the transition to their home environment with their SO (Legg & Penn 2013).

Individuals with ABI experience a loss of ‘pre-injury self’ which may involve a loss of independence, and loss of roles within the household (Chembeni & Nkomo 2017; WHO 2018). Upon discharge, individuals with ABI are likely to have inadequate activity and participation in their home and community because of physical or emotional limitations (Kusambiza-Kiingi et al. 2017). Complex emotions are often exacerbated as families can find it difficult to accept a new reality (Abrahamson et al. 2017; Piccenna et al. 2016; Villa, Causer & Riley 2020). Loss of friendships are likely to be experienced upon returning home as individuals with ABI express difficulty communicating with others (Chembeni & Nkomo 2017). This often leads to avoidance of familiar activities despite the need to participate within the home and community (Villa et al. 2020; Webster et al. 2015).

Social disconnect is another common experience as individuals with ABI may feel a loss of social status and experience limitations within their community (Soeker & Ganie 2019; Villa et al. 2020). Strain can be worsened in families when the ABI leads to communication difficulties (Ntsiea 2019). Families are often the most important support system while individuals with ABI recover and develop a new sense of self, reintegrating into home and community settings (Chembeni & Nkomo 2017; Villa et al. 2020; Webster et al. 2015). Understanding the lived experiences and emotional well-being in relation to the impact an ABI has on the individual and their SO has been continuously highlighted in research (Andersson et al. 2016; Harvey 2018). The need for quality information, communication and collaboration are emphasised as factors that reportedly would have improved an individual’s overall experience post-ABI (Andersson et al. 2016; Harvey 2018).

Experiences within the first month post-ABI are important because of various stages of rehabilitation to reinte-gration into communities (Abrahamson et al. 2017; Ntsiea 2019). Two of the highest-ranked needs during hospitali-sation involve clear explanations of the ABI and discussing realistic outcomes about recovery (Mauss-Clum & Ryan 1981). These needs continue to align with recent findings where individuals with ABI and SOs experience similar difficulties regarding the impact an ABI has on a family unit (Chembeni & Nkomo 2017; Holloway, Orr & Clark-Wilson 2019; Masuku et al. 2018; Villa et al. 2020).

The average stay in a South African tertiary level hospital is 6 days, and individuals with ABI are often discharged home too soon (Kusambiza Kiingi et al. 2017; Mudzi et al. 2012). Not all South Africans are covered by private medical schemes and therefore do not have easy access to rehabilitation facilities (Joosub 2019). In the public sector, few individuals with ABI benefit from post-acute rehabilitation because of an overburdened healthcare system and inadequate number of HCP to manage this caseload, limiting opportunities for them to access, adapt and learn about their recently acquired brain injury (Abrahamson et al. 2017; Joosub 2019; Taylor & Ntusi 2019). Transitioning into a home environment following ABI in an upper-middle income country, such as South Africa, has not been explored and further research is warranted.

Recent studies show that SOs experience emotional and physical strain while caring for individuals with ABI (Abrahamson et al. 2017, Masuku et al. 2018, Webster et al. 2015). Additionally, SOs often experience a lack of support from family members, likely exacerbating feelings of uncertainty, loneliness, and increased burden of care (Chembeni & Nkomo 2017; Webster et al. 2015.) Education and necessary referrals, while in acute care, may support SOs of individuals with ABI during the transition period (Harvey 2018; Holloway et al. 2019).

Previous studies from upper-middle income countries found communities and HCP may benefit from addressing the loss of emotional and physical independence post ABI (Bellon et al. 2015; Legg & Penn 2013). Home-based services are available in such cases, but referral pathways and implementation are not always adequate (Ntsiea 2019). In addition to home-based services, all families should have adequate access to information before discharge from acute care and while considering ongoing rehabilitation needs (Picenna et al. 2016). Another challenge within the public sector is premature discharge of individuals with ABI without adequate information about their recovery or available resources (Liang et al. 2017). This may result in complete dependence on family members (Ntsiea 2019). Research of this nature, conducted qualitatively, has proven the importance of examining experiences post-ABI (Abrahamson et al. 2017; Masuku, Mophosho & Tshabalala 2018; Souchon et al. 2020).

There are currently a few studies that focus on the transitioning periods from acute care to the community setting in upper-middle income countries, with low-income settings, such as South Africa (Kusambiza-Kiingi et al. 2017; Walker, Schlebusch & Gaede 2021; Webster et al. 2015). It is important that HCP base their decisions on real needs that are relative to the setting. Focusing on the current lived experiences of individuals with ABI and their SOs may highlight the importance of person-centered care and allow an easier transition to an adjusted way of living in future scenarios (Andersson et al. 2016; Harvey 2018). The aim of this study was to describe perceived experiences of individuals with ABI, and their SOs one-month post-discharge from acute hospitalisation.

## Research methods and design

The study aimed to achieve a deeper understanding of the direct perceptions or daily experiences of individuals with an ABI and their SO (Leedy & Ormrod 2015). Describing individuals’ experiences tied in with the theory of realism, which claims there can be different realities that exist based on the same situation, its meaning, and interpretations held by people (Creswell et al. 2016; Rahman 2017). Individuals’ contexts and experiences may influence their perceptions and beliefs (Maxwell 2012; Rahman 2017). This theory was appropriate for this study as it allowed for a better understanding of the relationship between individuals’ perspectives and their actual situations (Leedy & Ormrod 2015; Maxwell 2012).

### Research design and data collection

The twelve participants of this study were identified in two private, acute care facilities through the referral of the private Speech-Language Therapist (SLT) practice where the first author (K.T.) was employed. These two hospitals are in a South African city and offer services to outlying, smaller communities who do not have access to private hospitals in their hometowns.

Participants were interviewed one-month post-discharge from acute hospitalisation, focusing on experiences during hospitalisation and post-discharge. Informed consent was obtained by the first author (K.T.) during acute admission. Where this was not possible, the author emailed the necessary documents to the treating SLT at the rehabilitation facility. Two SOs who did not live nearby were also emailed consent and demographic forms for completion.

Purposive sampling was used to recruit six dyads of participants from each acute care facility (Leedy & Ormrod 2015). The first author made use of videoconferencing via WhatsApp video call as the primary medium to conduct a once-off semi-structured interview. The interview schedule adapted from Abrahamson and colleagues (2017) was used to probe detailed reasoning, making use of the real-time video, and audio feedback of the videoconference platform (Leedy & Ormrod 2015; Nehls, Smith & Schneider 2015).

Minor adjustments were made to the previously published interview schedule to include questions about discharge from hospital and transfers to step-down and rehabilitation facilities (Bellon, Kelly & Fisher 2021; Liang et al. 2017; Whitehead & Baalbergen 2019). Questions were also adapted to be open-ended and original questions were split into different parts to obtain more information from the participants (Leedy & Ormrod 2015; Schonlau et al. 2019). A desktop computer (Intel Core i7-8700K CPU @ 3.70GHz, 32GB RAM), Apple iPad Pro (3^rd^ Generation, 12.9”, 256GB, Wi-Fi) and webcam (Trust Spot Light Pro Webcam) were used to conduct and audio-record interviews. The interviews were then uploaded to a website (Otter.ai n.d.) for automatic transcription. Transcripts were manually edited by the first author [KJT]. Communication supports were not used during interviews as all participants could effectively verbalise opinions without external support. The interviews lasted an average of 30 minutes. One participant showed signs of fatigue as the first author made use of most of the guiding questions for more detailed responses.

### Participants

Participants were only included once a diagnostic CT (computerised tomography) or MRI (magnetic resonance imagery) scan confirmed the presence of a single ABI and subsequent referral to SLT services during acute hospitalisation. Upon administering the Cognitive-Linguistic Quick Test (CLQT) as a routine assessment, individuals with ABI who scored between 2.5 – 4.0 on their overall composite severity rating (CSR) and ‘mild’ or ‘within normal limits’ on their language abilities, were considered for inclusion (Box 1). All participants had to be older than 18 years and have conversational English. Once the individual with ABI was invited to participate, their nominated SO was also invited to take part, forming a dyad. The SOs were only included if they were primarily responsible for caring for, and lived with or near, the individual with ABI (Box 2). Participants were recruited between February and July 2021. A pilot study was conducted with one dyad, but these data were not included in the final sample because of changes that were made to the interview schedule subsequent to the pilot study.

BOX 1 Inclusion and exclusion criteria for participants with an ABI.

**Table:** 

Inclusion criteria	Presence of a single ABI, confirmed by a medical report or results of a CT and/or MRI scan, interpreted by the radiologist, Participant was aged 18 years and older, Participant was referred to the SLT during acute hospital admission for treatment, Participants used English as one of their more dominant languages, or using it with ease (Kiran & Gray 2018) Participants who could provide verbal or written consent, Participant could participate in verbal conversation, express basic needs, and produce short sentences (Abrahamson et al. 2017). This was established by the treating SLT Participants’ CSR for their cognitive abilities fell between the ranges of 2.5-4.0 on the CLQT. The translated scores indicated cognitive abilities ‘within normal limits’ (WNL) or ‘mild’, Participants’ SR for their language abilities fell within the ranges of ‘mild’ to ‘WNL’ on the CLQT, Participants fell within the time period of one-month post-discharge from an acute hospital setting, Participants had access to a smartphone with the WhatsApp application downloaded, or alternatively Zoom.
**Exclusion criteria**	Participants were excluded if they had severe communication and/ or cognitive impairments. This was based on whether the person with the ABI was able to engage in meaningful conversation or if there were concerns about their capacity to provide consent, Participants who had co-occurring diagnoses such as dementia or Alzheimer’s disease, that may influence results, were not asked to participate.

ABI, acquired brain injury; CT, computerized tomography; MRI, magnetic resonance imaging; SLT, speech-language therapist; CSR, composite severity rating; CLQT, cognitive-linguistic quick test; WNL, within normal limits; SR, severity rating.

BOX 2 Inclusion and exclusion criteria for SO of the individual with an ABI.

**Table:** 

Inclusion criteria	Participant was a partner, carer, family member or friend of the person with the ABI (Hatteberg 2020), SO lived with, or was in a position to visit regularly (at least twice a week) with the person with the ABI, SO had an ongoing relationship with person with an ABI, Participant provided direct care to the person with an ABI (e.g., bought their groceries or took them to their appointments), Participants were aged 18 years and older, Participants used English as one of their more dominant languages, using it with ease. (Kiran & Gray 2018) Participants had access to a smartphone device with the WhatsApp mobile application, or alternatively Zoom
Exclusion criteria	Participants were excluded if the person with the ABI did not consider the person as their significant other, Participants were excluded if they were not directly involved in caring for the individual with ABI.

ABI, acquired brain injury; SO, significant other.

The six participants with ABI were aged between 19-57 years old, and the SOs were 42-48 years of age. Five participants were male and seven were female (Table 1). The six SOs were directly related to the individuals with ABI and had familial roles of daughter, wife, husband, mother and sister. The youngest participant was a student who could not continue with her studies, and one participant opted for early retirement, aged 47 years old. As a result of their ABI, the remaining four participants required assistance returning to work. Ten participants were permanently employed. In addition to the SOs’ own employment, there were demands to assist in running their family members’ business post-ABI. Table 1 further elaborates on participants’ employment. Participants’ home languages included Afrikaans, Northern Sotho, and Southern Sotho. None of the participants spoke English as their primary home-language but used it conversationally. Interview questions were therefore repeated when necessary, simplified to use layman’s terms and the first author translated to obtain the appropriate English word from Afrikaans participants, when asked. None of the individuals with ABI could return to driving after their injuries.

**TABLE 1:** Participant description – Individuals with ABI (*n* = 6) and their SO (*n* = 6).

Participant with ABI	Age	Gender	Home Language	Employment Status	Reason for admission	Condition prior to incident	Participant: SO	Age	Gender	Home Language	Employment Status	Relation to the person with an ABI
P01	56	M	Afrikaans	Full time, Self-employed	TBI – fell from bicycle	Independent	SO01	42	F	Afrikaans	Full time, Self-employed	Daughter
P02	47	M	Northern Sotho	Early retirement	TBI - Assault to the head	Independent	SO02	42	F	Southern Sotho	Full time, Paid employment	Wife
P03	19	F	Afrikaans	Student	TBI - Motor vehicle accident	Independent	SO03	47	F	Afrikaans	Full time,Paid employment	Mother
P04	57	M	Afrikaans	Full time, Self-employed	CVA	Independent	SO04	48	F	Afrikaans	Full time, Self-employment	Wife
P05	46	M	Southern Sotho/ English	Full time, Paid employment	CVA	Independent	SO05	43	F	Southern Sotho and English	Full time, Paid employment	Younger sister
P06	37	F	Afrikaans	Full time, Paid employment	CVA	Independent	SO06	43	M	Afrikaans	Full time, Paid employment	Husband

ABI, acquired brain injury; TBI, traumatic brain injury; CVA, cerebrovascular accident (stroke); SO, significant other; F, female; M, male.

### Data analysis

Data were analysed using inductive thematic analysis to allow for data to be coded without placing results into a pre-existing coding framework (Braun & Clarke 2013). Thematic analysis aimed to examine participants’ experiences and perspectives in a direct manner, without assuming any other underlying meanings (Braun & Clarke 2013; Leedy & Ormrod 2015).

The first author read through and manually corrected the transcriptions against the audio recordings. Thematic analysis followed steps outlined by Braun and Clarke (2013). The first author initially read through the transcripts to identify common experiences, which was a key phase when interpreting the qualitative data set (Bird 2005). Experiences focused on preparation for discharge, services received during admission, adapting from acute care and transitioning back to social and home environments, one-month post-discharge from acute hospitalisation. The second phase involved using Atlas.ti 9 software (Scientific Software Development GmbH 2020) to establish codes and subsequently highlight similarities and differences between the data sets of individuals with ABI and their SOs (Braun & Clarke 2013). The common experiences were highlighted and grouped together.

Through revisiting the raw data and debriefing with co-authors, consensus on themes was reached in phase three (Braun & Clarke 2013; Elliot 2018). Sub-themes were created where similar ideas could be grouped and discussed together. Discussion among the three authors contributed to credibility of the data and provided alternative suggestions where necessary (Connelly 2016; Leedy & Ormrod 2015). This ensured that the interpretation and development of themes reflected the participants’ personal experiences and related to the aim of the study.

### Ethical considerations

Ethical clearance to conduct this study was obtained from the University of Pretoria Faculty of Humanities Research Ethics Committee (No. HUM039/0920).

## Results

**FIGURE 1:**
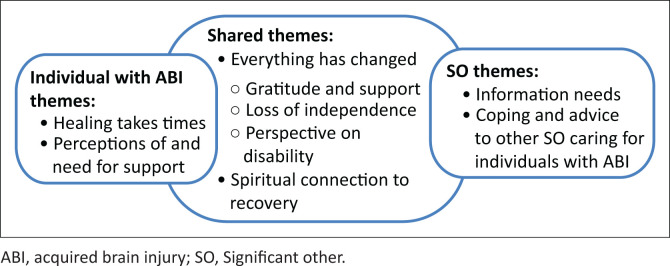
Themes that emerged based on participants’ personal experiences.

Six main themes were agreed upon after analysing the personal experiences of the individuals with ABI as well as their respective SOs (Figure 1). Between the two groups, two themes were shared. One of the shared themes developed three sub-themes. Fictitious names were used for participants and hospitals.

Interviews took place during the COVID-19 pandemic, exacerbating existing stresses surrounding the onset of an ABI. The pandemic negatively influenced all participants’ well-being because of the changes in hospital regulations and protocols. All dyads commented on the difficulties coping while not being able to visit family and see familiar faces regularly within the hospital and rehabilitation settings. The resultant themes discussed are thus reflective of the circumstances surrounding the COVID-19 pandemic.

## Shared themes to both individuals with acquired brain injuries and their significant others

### Everything has changed

The first shared theme discusses experiences of the individuals with ABI adapting to a new reality, losing independence, and as a result, their SOs now taking on the role of caregiver. All individuals with ABI acknowledged changes to their physical abilities and anticipated changes to participation in everyday activities. P04 stated: “My whole life is going to change when I’m going back home.” Although the mentioned consequences of the ABIs were mainly physical, emotional consequences were also perceived. Emotional well-being was important to P03 as conveyed in this statement: “Emotionally I want to be okay.”

Contrastingly, two of the six individuals with ABI felt expectations from their SOs and those around them: ‘Everybody just expects me to be okay.’ (P03, age 19, female)

SOs had difficulty adjusting to the new role as caregiver towards their family member with ABI and expressed a clear view on their increased burden of care and consequent emotions. SO01 suggested the following as a result of her experience:

‘Don’t take everything on one person because that’s a huge burden to carry.’ (SO01, age 42, female)

Emotional challenges around coping with the changes post-ABI in the loved ones of the six SOs that were interviewed were highlighted:

‘Well I had to get prescription drugs to keep me calm… Taking it [*effects of an ABI*] like literally day by day. Ja, that’s how you do it. You [*SO*] go day by day.’ (SO01, age 42, female)

In addition to emotional challenges, individuals with ABI were disheartened that they could not return to their usual social activities such as singing lessons, golf and karate. Three individuals with ABI expressed concerns regarding their communication, ability to focus, and memory:

‘I cannot explain myself sometimes … Just difficult for me to speak the words I’m used to speaking you know. Even with English, Afrikaans, it’s the same.’ (P01, age 56, male)

‘I struggled so much to concentrate. To focus.’ (P03, age 19, female)

These difficulties directly influenced their experience within the workplace (P01) and progress within the rehabilitation hospital (P03). Most individuals with ABI expressed feelings of physical and cognitive fatigue, which was new to them.

#### Gratitude and support

Although the change post-injury brings challenges, individuals with ABI expressed appreciation for what they could do prior to the injury and to have survived. P03 was grateful for the opportunity to share with other patients and learn from their experiences:

‘We also talked a lot about how grateful we are about where we are in our lives, alive… We learn from each other. We speak to each other. This is what I’m grateful for, this is what I’m going through, things like that. It’s also great to experience that [*sense of community*] because if you had to just lie there, you feel sorry for yourself.’ (P03, age 19, female)

One participant expressed appreciation for previous abilities and the realisation of the loss thereof:

‘If somebody told me I can choose ten million rand or walking, I will take the walking.’ (P04, age 57, male)

SOs also reflected on their family member with an ABI and the appreciation felt for what they have done for their family prior to the ABI as mentioned by a participant:

‘I understand him better now, how busy he is on the farm … So yes, I’m appreciating him very much now.’ (SO04, age 48, female)

One participant discussed the importance of patience and education regarding ABIs. He was appreciative of the healthcare team that took time to explain the causes of stroke, and prevention thereof, which was important to share with those around him. As a result of the education, P06 chose to change his lifestyle as a precautionary measure:

‘The only thing is I have to take care of myself. And like I say I don’t want to drink anymore. I think it’s taking care [*of himself*] is the most [*important*] thing stroke has taught me.’ (P06, age 37, female)

#### Loss of independence

All participants were disheartened as they became dependent on their SO for at least one activity of daily living, such as driving, cooking, and bathing. One participant admitted to this by stating:

‘She’s [*SO*] also doing things that she didn’t do before … She’s cooking, she’s doing everything for me.’ (P02, age 47, male)

P02 and P03 reported difficulty with their vision and hearing at the time of their interview. These difficulties interfered with activities such as shopping, leisure activities and mealtimes:

‘My ears were so sensitive to noise … my mom was busy chewing an apple and I was busy filling in a wordsearch. I couldn’t concentrate.’ (P03, age 19, female)

None of the six individuals with ABI could drive post-discharge. The participants were concerned regarding their loss of independence:

‘They [*medical doctor*] told me for about six months that I won’t be able to drive. So that’s also gonna be a little bit of an adjustment because I’m used to being independent. I don’t normally ask someone to take me somewhere to do grocery shopping or things like that.’ (P06, age 37, female)

P02 attempted driving but reported difficulties with his vision and did not resume driving thereafter:

‘When I’m driving [*in the car*] there are four [*pictures*] of the road. I can meet up in an accident very easily.’ (P02, age 47, male)

Regarding SOs, three were already adapting to living with their family member with an ABI, while the rest were awaiting their family member’s discharge from a rehabilitation facility. SO01, SO02 and SO05 expressed challenges caring for someone who is no longer as independent as before. SO02 experienced additional struggles when it came to bathing her family member:

‘I will go with him in the bath. I must get in the bath. I must sit by. I must give him the face cloth. It’s like I’m teaching a 6-year-old to wash. I’m struggling because we don’t know how long he is going to be like this [*dependent*].’ (SO02, age 42, female)

Unlike the other dyads, SO05 only expressed an increased role of driving her brother to appointments and for running errands as the major change:

‘That’s the only thing that has changed. I think I’m the chauffeurchauffeur.’ (SO05, age 43, female)

Four of the six individuals with ABI could not return to work post-discharge. These four participants understood the reasons for not returning to work and were willing to make necessary arrangements to ease into work settings as their recovery allowed. The other individual with ABI (P03) was a student who could not return to her undergraduate studies, and the last participant was on early retirement. As a result of her ABI, P03 was devastated that she could not return to her studies as planned:

‘I thought I would just rest the week and go back to Bloemfontein and study again, and it was not the case. Every time it [*feedback from HCP*] was just, okay you cannot do this, you cannot do that … and the saddest part for me was the fact that I couldn’t study this year again.’ (P03, age 19, female)

Work-related activities were also discussed as SO01 spoke about loss of independence in the workplace:

‘He [*family member with ABI*] still has his own business which he can’t manage, which I’m managing now … He has not very good short-term memory … And now somebody else must step in for everything that he did and is not doing now.’ (SO01, age 42, female)

All six SOs anticipated or admitted to needing additional help at home because of the uncertainty of the ABI. In addition to the increased burden of care, two SOs expressed feelings of guilt regarding the ABI of their family member. Their statements were as follows:

‘Nothing on earth will actually prepare you for this [*ABI*] … If she [*family member with ABI*] listened, this wouldn’t have happened. So now I’m asking you, how do we deal with this guilt?’ (SO03, age 47, female)

‘It’s [*caring for someone with ABI*] not easy. Sometimes it feels as if you are the cause of that [*challenges experienced since the ABI*].’ (SO02, age 42, female)

#### Perspective on disability

Another concern among the SOs was that of stigma associated with ABIs. SO03 was comfortable sharing about the ABI with others whereas the individuals with ABI did not have similar feelings or attitudes:

‘She [*family member with ABI*] is cross with me because she doesn’t want people to know that she had a brain injury. How must I lie about that?’ (SO03, age 47, female)

SO02 acknowledged stigma and ways to overcome it, creating a positive environment for her and her husband with an ABI:

‘He mustn’t feel like because now that he is having this injury, … he has this scar at the back of the head, now we don’t even want to take him to the people. Like ‘you must always stay at home’, ‘You mustn’t come with us.’ No! You must comfort him. You must be the same with them [*individuals with ABI*].’ (SO02, age 42, female)

A person’s culture may guide their overall experience. SO05 recollected how the implications of the ABI were more difficult based on how gender is perceived in her culture:

‘Remember, in Zulu culture, the male figure, they call them the prince. They’re the most important people in the family. So, it was a very tough situation when he was very sick.’ (SO05, age 43, female)

Three family members experienced positive outcomes resulting from the ABI, including their family member becoming motivated about leading a healthy lifestyle. One SO expressed appreciation for her husband’s job and hard work since taking over his business, as is attested to in the following statement:

‘I understand him [*husband with an ABI*] better now, how busy he is on the farm. Really busy. And what he has and is doing for us. Because it’s really a 24-hour job. He is the whole time busy… So yes, I’m appreciating him very much now. We are 29 years on the farm and now I know what he has done all these years.’ (SO04, age 48, female)

### Spiritual connection to recovery

Three individuals with ABI acknowledged and were grateful for their spirituality during their recovery. P04 boldly shared: ‘But now God has given me a second chance and therefore I must rise for that… I have to fight for my wife and my kids,’ and remained motivated throughout treatment as a result.

P03 commented on her “second chance” when discussing the severity of the car accident, ‘I couldn’t believe that I walked out of there alive. I got a second chance. I can’t even compare myself to the person I was a few weeks ago.’

Spirituality was tied into experiences that involved healing:

‘After 24 hours, they were supposed to declare me dead. I got through the 24 hours to 33 days, and I woke up … My ancestors were with me.’ (P02, age 47, male)

P02 further offered advice to other individuals with ABI that involved trusting (higher power) and letting (this higher power) fight for you through the tribulations. It was evident that this participant found encouragement through spirituality.

Three of the six SOs shared their spiritual beliefs and the reliance on faith as integral in managing day-to-day challenges. SOs discussed how prayer helped them cope through the difficult period and the perceived premonition that warned them of something that was about to happen to their family member. SOs appeared to be thankful for their spirituality as it helped their family member with ABI through the healing process. SOs were aware of medical intervention surrounding the ABI, but still relied on their faith. The following attests to this:

‘Even though they’re [*HCP*] not sure whether he [*family member with ABI*] will be hundreds, but everything is going to be fine as we are hoping. All of us hope for the best. Only God provides.’ (SO05, age 43, female)

## Themes of participants with an acquired brain injury

### Healing takes time

Five of the six individuals with ABI were transferred to a rehabilitation facility after discharge from their respective acute hospital. They acknowledged the effects of their ABI as more serious than they expected and, consequently had extended hospital stays for rehabilitation:

‘The repercussions of this thing [*head trauma*] is like, giant … I didn’t know that this would take so long for everything [*cognitive-communication skills*] to come back and heal … I was in the hospital more than I was home this year.’ (P03, age 19, female)

Along with the consequences of the ABIs, all participants were aware of the time factor that partnered with healing and the patience to regain independence. P06 shared about her progress:

‘I know it might sound maybe cliché, but I think it’s better to be positive, than to be negative. And the quicker that you get into it, to accept that you need help, you will do better… Like in the start, I couldn’t take a ball, just a small ball to put it from one bucket to another one. And now I’m using my toothbrush.’ (P06, age 37, female)

Two of the three individuals with ABI (P01 and P05) who were discharged at the time of their interview were attending outpatient therapy. P01’s statement showed awareness of his additional therapy needs:

‘I was going out of the hospital, and I heard I’m coming back every second week. Then I started realising that this [*communication*] is actually the main reasons.’ (P01, age 56, male)

Although P02 was not attending out-patient therapy, he continued to exercise at home to return to previous activities:

‘I must not stay in one place. I must go up and down just a little bit in the house so that I must get used to those things [*exercise*] again.’ (P02, age 47, male)

An increased awareness of the individual with ABI’s limitations appeared to be motivating and created a positive experience when noticing their own progress.

### Perceptions of and need for support

All individuals with ABI expressed their emotions towards their injury were suppressed as they tried to cope and recover. While participants had differing opinions of how positive their hospitalisation was, they all expressed a need for specific support. The perception of their need for support may have been influenced by the context of the COVID-19 pandemic and changes in hospital staffing patterns during the time of crisis.

Participants perceived a need to have a space for therapeutic sharing. They might have had difficulties with the ABI itself, but the need that was expressed was one of sharing the experience that they have had, as is shown by P03:

‘Everybody knows what I’ve been through, but nobody knows how I felt.’ (P03, age 19, female)

Two of the six ABI survivors expressed benefits of sharing thoughts and feelings with others who had also experienced such an injury. Services offered by social workers and psychologists were available to all individuals in this study, but only three made use of them. Participants suggested that acute and rehabilitation facilities consider creating environments for individuals with ABI to share experiences, such as support groups, which encourage emotional and psychological progress:

‘I was talking to everybody and I was starting to feel better’ (P01, age 56, male)

‘They [*The rehabilitation facility*] should implement something where they can help people to be more positive about their situation’ (P06, age 37, female)

While some participants were positive about their encounters in the facility, others had negative experiences. Some participants raised complaints regarding service provision and readiness for discharge. Two individuals with ABI expressed that nursing staff were not proactive in looking after them during acute hospitalisation. Additionally, P04 was upset because he felt that he did not receive individualised care in the rehabilitation setting:

‘So, some days they [*physiotherapists*] leave you for a moment to take care of another patient. I don’t like that because I want them to finish with me before going on.’ (P04, age 57, male)

This participant voiced a misalignment in the support he received and what he felt he required. The feeling of frustration was evident as P04 became flustered discussing care within the facilities, losing grip of his cellphone, and requiring assistance from care workers during the videoconference.

Despite the expressed need for improved support, there was also a perception that their experience was mostly favourable. Three participants were still hospitalised in a rehabilitation unit, subsequent to discharge from an acute hospital, at the time of their interview and reported positive experiences regarding progress made. Two of the three participants felt adequately prepared to return home despite challenges they may still face:

‘The therapists and the doctor are also giving me so much information. I feel better about all the anxiousness and … going home.’ (P06, age 37, female)

Participants acknowledged the impact of rehabilitation and took note of their own progress made during this time. Two participants shared their experiences:

‘This place [*rehabilitation facility*] did wonders for me really.’ (P03, age 19, female)

‘My brain got better when I was here at Hospital H.’ (P04, age 57, male)

## Themes of significant others

### Information needs

Among all SOs, a clear need arose for streamlined services by HCP in the hospitals, as well as education and written information presented during the acute stage. As a result of poor coordination of services, there was limited guidance offered to SOs at the time of discharge:

‘No one told me that [*individual with ABI*] needs rehabilitation. No one’ (SO03, age 47, female)

Another statement regarding realistic expectations was also made by SO03,

‘Sometimes she gets aggressive, and I don’t know why because they didn’t even tell me you must expect something like this.’ (SO03, age 47, female)

Written information regarding various rehabilitation facilities and services would have been helpful in the case of SO01 where she suggested:

‘I suggest brochures … because now everybody is phoning you with no concrete anything. I never took down anything over the phone. I told them, mail me, so I can read through it.’ (SO01, age 42, female)

It appears that participants preferred information in a written format, hard and/or electronic copies, to make informed choices. Information being provided verbally or telephonically may have contributed to the overwhelming emotions felt at the time of transfer from the hospital to rehabilitation facilities. The COVID-19 pandemic may have further contributed to this view as family members could not visit the hospital or rehabilitation facility and HCP may have relied on telephonic means of communication.

The importance of information to guide decision making was highlighted in the following statement:

‘I did agree to anything they [*HCP*] were saying because I just assumed … they’re professionals, they won’t do something that was going to hurt him [*family member with ABI*]’ (SO02, age 42, female)

When the opportunity arose for the SOs to ask questions in the rehabilitation facilities, they felt informed and satisfied with what they were told. This opportunity only arose in the rehabilitation facilities when family meetings were held and not in the acute settings.

Improved communication between SOs and HCP would have improved the overall experience transitioning from acute care. SO01, SO04 and SO06 perceived communication with HCP in the acute setting as poor. The following statements attest to this finding:

‘Nobody phoned us for about four days’ (SO01, age 47, female)

‘It must be easier to communicate with them [*hospital staff*].’ (SO04, age 48, female)

‘You don’t know what’s going on. Really.’ (SO06, age 43, male)

### Coping and advice to other significant others who are caring for an individual with an acquired brain injury

All SOs offered advice or suggestions on how to cope when a family member has an ABI. SO06 and SO02 respectively stated the following which portrays positivity and a sense of embracing their new situation:

‘You can totally change your life. It doesn’t mean it’s a life sentence. She [*individual with ABI*] is going to be more motivated about her lifestyle than in the past.’ (SO06, age 43, male)

‘We must accept the situation and they [*families members caring for individual with ABI*] must also give the person [*with an ABI*] love.’ (SO02, age 42, female)

Two SOs had an idea of what to expect and felt better prepared as they had previous experience caring for a family member with an ABI:

‘I have a disabled sister … And I know what she does. And when I saw this, I could make the similarity.’ (SO03, age 47, female)

‘My dad couldn’t speak … So, I know what is happening now.’ (SO04, age 48, female)

Importance of family support was emphasised and reliance on family members was encouraged by SO01, SO02 and SO05. There is a clear benefit in cases where other family members are also able to assist in full-time care:

‘She’s [*individual with ABI*] staying at my mother-in-law at this stage. She’s [*mother-in-law*] always at home, and she can drive and everything … it’s helping me and her [*individual with ABI*] because now she doesn’t feel she can’t do anything for herself, because she can still move like she wants to because her mom is with her … Like I said, I’m working the whole time. If she was staying at home, she would get lonely.’ (SO06, age 43, male)

## Discussion

The six main themes provide insight into the lived-experiences of a sample of individuals with ABI and their SOs one-month post-discharge from an acute facility. Their experiences were largely influenced by the COVID-19 pandemic. Improved communication with staff, the need for written information, a lack of physical contact with loved ones, a need for support groups as well as more streamlined services, were factors that shaped participants’ experiences.

All individuals with an ABI expressed that emphasis was placed on making progress with physical abilities and minimal opportunities arose to work through their emotional trauma. Feedback within peer support groups may be beneficial for individuals with ABI to monitor their progress and emotional well-being (Reese et al. 2009). Support groups and psychosocial treatment can be encouraged in the early stages of care by allied HCPs (Nash et al. 2021; Wijekoon 2020). Not all participant experiences were negative. Participants additionally offered advice to other individuals with ABI in earlier stages of recovery, who may experience similar situations in the future. Sharing experiences and making sense of recovery between fellow survivors with an ABI may provide support in ways that are different to that of HCP and family members (Kersten et al. 2018; Wijekoon 2020).

A loss of an individual’s idea of self, pre-injury, may alter how they perceive themselves within a familiar context, post-ABI, as they can no longer participate in tasks as actively or independently as before (Chembeni & Nkomo 2017). Counselling is important and likely provides means for individuals with ABI and families to cope with stigma, loss and other emotional challenges post-ABI (Joosub 2019). Although beneficial, psychosocial intervention may not always be available or affordable, in upper-middle income countries such as South Africa (Harrison et al. 2017; Joosub 2019; Pillay & Barnes 2020).

Service provision, education and readiness for discharge were concerns raised by individuals with an ABI. Sufficient preparation and education around the time of discharge may ease anxiety and uncertainties felt post-discharge from acute hospitalisation (Abrahamson et al. 2017; Walker et al. 2021). Unfortunately, the transition between acute and rehabilitation facilities has been reported as fragmented in some instances (Piccenna et al. 2016). Healthcare teams could improve their handover by including a written report to HCP at the receiving facilities for improved continuity of care. This may allow for better understanding of the individual’s current level of functioning and abilities transitioning from the acute hospital. Readiness for discharge can be determined by a team of collaborative professionals to ensure the best outcomes for the individual with an ABI and their family (Walker et al. 2021).

The two individuals with ABI who felt adequately prepared for discharge had improved insight into their ABI and were aware of ongoing rehabilitation needs. This reflects positively on patient education and person-centred care (Berntsen, Yaron, Chetty et al. 2021). Allied HCP, such as SLTs, can create opportunities for individuals with ABI to ask questions, improving service provision along the continuum of care, through education (Gauvreau & Le Dorze 2020; O’ Connell et al. 2021).

In addition to health education, participants appear to rely on their faith during challenging times. Corroborated by other studies, individuals with ABI also mentioned their belief being an important aspect of the recovery process (Karpa et al. 2020; Masuku & Khoza-Shangase 2018; Souchon et al. 2020). Faith and spirituality were the centre of most of participants’ motivation and recovery.

Emotional well-being was important to individuals with ABI as they often felt misunderstood by their families and peers. Although motivated by progress, individuals with ABI remained anxious about activities such as driving and returning to work (Kusambiza-Kiingi et al. 2017; Walker et al. 2021). There are recent reports of South African companies offering additional support to individuals with ABI readjusting to the workplace (Akbar & Wissink 2018). An implication of this study would be for more companies to offer such benefits for individuals with ABI easing back into the work setting. Where this is not possible, psychosocial support and rehabilitation is re-emphasised (Whitehead & Baalbergen 2019). Neuropsychological difficulties in certain areas of functioning may influence the return to productive work and consistent neuropsychological assessment is highlighted (Fortune et al. 2021).

A loss of function in individuals with ABI may result in increased burden of care on the SOs (Bordonada 2017; Kreutzer 2018). In this study, all participants were fortunate to have the option of both in- and out-patient treatment, however this is not always the case for the general South African population (Joosub 2019). High-caseloads and time pressures, with few qualified allied HCP, could have led to disparities in access to care (Nash et al. 2021). SOs try to navigate changes in their loved ones as well as their own new roles and responsibilities. HCP can be a vital source of support to SOs at the beginning stages post-ABI, and throughout the rehabilitative process (Cheklin et al. 2020). Trained caregivers who offer additional psychosocial support have been regularly researched and encouraged in the literature (Karpa et al. 2020; Kreutzer 2018). One of the SOs caring for the individual with ABI at home felt encouraged and empowered by the preparation received from the step-down facility.

In this study SOs were willing to share their knowledge about ABIs to their communities, utilising the opportunity for education. Culture is likely an important factor that may influence how stigma is expressed and experienced by people living with disabilities and there is a need to move beyond traditional ideas to create awareness, but also encourage belonging within communities (Jansen-van Vuuren & Aldersey 2020). SOs expressed that being positive and embracing difficulties will help the family heal, as was found previously (Lond & Williamson 2019). On the contrary, the individuals with ABI were more self-conscious of their diagnosis and its implications. Increased self-awareness could be because of the stigma of an ABI (Villa et al. 2020). Access to helpful and accurate information about various diagnoses may be hindered because of misinformation, or cultural beliefs and practices (Villa et al. 2020). Future research studies could investigate spirituality and culture and the influence on individuals’ attitudes to healing. Healthcare professionals such as SLTs, can address this need for education through peer-based support groups. Health literacy is important for families to better understand injuries and carry information into communities of any context (Li et al. 2020).

A finding of this study was the important role that the COVID-19 pandemic played in shaping individuals’ experiences. The pandemic caused great upset despite the HCPs attempts of consoling patients and families, while adapting to the hospitals’ changing protocols (Aquila et al. 2020). Emotional support and mental health of patients and HCPs were important during this time, but the need may not have been addressed, possibly influencing the quality of engagement between HCP, individuals with ABI and their families (O’Connell et al. 2021). Further research is warranted in this area.

The most prominent difficulty caused by the pandemic was cancellation of hospital visiting hours. This likely resulted in HCP filling familial roles, simultaneously attempting to maintain a professional boundary (Aquilia et al. 2020). Visiting hours provide an opportunity for HCP to give feedback to families, subsequently benefiting the individual with an ABI (Silvera, Wolf, Stanowski et al. 2021). By terminating face-to-face interactions, HCP most likely adjusted to using electronic communication (Boulton et al. 2021). This may have contributed to the negative experience regarding communication felt by SOs in this study.

### Limitations of current study

Although this study consisted of a small sample of 12 individuals, it yielded clinically relevant findings that could be useful for HCP during the acute stage of recovery. Lived-experiences post-discharge have been widely explored, but there is limited research focusing on personal experiences one-month post-discharge from acute hospitalisation in settings such as South Africa (Souchon et al. 2020; van Zyl et al. 2019).

An interpreter was not used for this study, narrowing the responses to include only English, which was none of the participants’ first language. A longer time frame post-ABI may allow for more experiences to be shared. The first author only included individuals with ABI who had mild cognitive-communicative difficulties, excluding experiences of individuals with moderate to severe ABI. To represent the diversity of the South African population, further large-scale studies in both public and private health sectors could be undertaken.

## Conclusion

Effective communication, health education and handover between the healthcare team, individuals with ABI, and their SOs could improve individuals’ overall experiences in the acute stages of recovery post ABI. As a result of subsequent emotional challenges, psychosocial intervention and support groups should be encouraged and made standard practice in acute and rehabilitation facilities (Panday et al. 2021).

Improved person-centred intervention and an established continuum of care may assist individuals with ABI and their families transition from hospital to their home environments (Whitehead & Baalbergen 2019). It may happen that individuals with ABI are discharged and unable to return for follow-up services (Joosub 2019). Clear professional communication, teamwork, and collaboration is paramount during the acute stage of care. The SLT and other HCP could therefore effectively use the time in hospital to ensure the most pertinent information is conveyed. Research on the use of therapeutic support groups and counselling techniques should be further explored within healthcare systems similar to that of South Africa such as Botswana and Namibia (Legatum Institute 2021).
